# Prevalence and Spectrum of Chronic Liver Disease Among Patients Seeking Health Care in Ghana

**DOI:** 10.1111/liv.70538

**Published:** 2026-02-14

**Authors:** Felix Lehmann, Alexander Killer, Sarah Wels, Stefan Schmiedel, Richard Odame Phillips, Pia Luise Roppert, Kirsten Alexandra Eberhardt, Martha Charlotte Holtfreter, Sabine Stauga, Ansgar Wilhelm Lohse, Stephan Ehrhardt, Ohene Opare‐Sem, Hans Martin Orth, Fred Stephen Sarfo, Christian Drosten, Anna Maria Eis‐Hübinger, Tom Luedde, Dieter Glebe, Jan Felix Drexler, Torsten Feldt

**Affiliations:** ^1^ Institute of Virology, Charité ‐ Universitätsmedizin Berlin Corporate Member of Freie Universität Berlin and Humboldt‐Universität zu Berlin Berlin Germany; ^2^ Department of Gastroenterology, Hepatology and Infectious Diseases, Medical Faculty and University Hospital Düsseldorf Heinrich Heine University Düsseldorf Germany; ^3^ Department of Infection Control, Hygiene and Environmental Medicine Public Health Authority Städteregion Aachen Aachen Germany; ^4^ Division of Infectious Diseases, First Department of Medicine University Medical Center Hamburg‐Eppendorf Hamburg Germany; ^5^ Infectious Diseases Clinic University Medical Center Hamburg‐Eppendorf Hamburg Germany; ^6^ Directorate of Medicine Komfo Anokye Teaching Hospital Kumasi Ghana; ^7^ Institute of Medical Virology, National Reference Center for Hepatitis B Viruses and Hepatitis D Viruses Justus Liebig University Giessen Germany; ^8^ Bernhard Nocht Institute for Tropical Medicine Hamburg Germany; ^9^ Department of Epidemiology Johns Hopkins Bloomberg School of Public Health Baltimore Maryland USA; ^10^ Institute of Virology University Hospital Bonn Bonn Germany

**Keywords:** chronic liver disease, Ghana, hepatitis B virus, transient elastography, viral hepatitis

## Abstract

**Background:**

Chronic liver diseases (CLD) leading to liver fibrosis and cirrhosis are a major cause of morbidity and mortality in sub‐Saharan Africa and pose a significant burden on its health care systems. We aimed to elucidate the prevalence of fibrosis/cirrhosis in patients seeking health care in Kumasi, Ghana, and its underlying aetiologies.

**Methods:**

In this cross‐sectional study, we performed sonography, transient elastography as well as biochemical and virological analyses.

**Results:**

Transient elastography indicated fibrosis/cirrhosis in 24.5% (113/461) of participants. Liver cirrhosis was significantly associated with known hepatitis B virus (HBV) infection, lack of formal education, hospitalisation, and male sex. Prevalence of active hepatitis B was significantly higher in patients with liver cirrhosis compared to controls (54.6% [30/55] vs. 17.1% [19/111]), as was anti‐HBc (94.6% [52/55] vs. 80.2% [89/111]). CLD was mainly attributed to HBV (27.3%, 30/110), alcohol abuse (11.8%, 13/110), a combination of both (10.9%, 12/110), and metabolic dysfunction‐associated steatotic liver disease (MASLD) (20%, 22/110). Antiviral treatment was indicated in 24 patients with active hepatitis B (number‐needed‐to‐screen: 19.2). Hepatitis C and D viruses were of minor importance (2.7% [3/110] and 0.9% [1/110], respectively).

**Conclusions:**

We found a high prevalence of CLD, predominantly caused by HBV, MASLD and alcohol. We confirmed the use of transient elastography as a non‐invasive and easily applicable tool in resource‐limited settings. Our findings underscore the need for systematic screening of hospitalised patients, especially men, in sub‐Saharan Africa. Comprehensive screening, treatment, vaccination and prevention programs for HBV, as the leading cause of chronic liver disease, are warranted.

## Summary

- Chronic liver disease can lead to liver fibrosis and cirrhosis and has a strong impact on the healthcare systems in sub‐Saharan Africa, a resource‐limited region.
- In this study, we used a non‐invasive method to screen for chronic liver disease in people seeking health care in a Ghanaian hospital, which revealed that a quarter of study participants had indications for either liver fibrosis or cirrhosis.
- We found evidence for either non‐alcoholic fatty liver disease, hepatitis B virus infection or heavy alcohol consumption as apparent underlying causes in more than two thirds of all patients with chronic liver disease.

**Abbreviations**
A1AT: alpha‐1 antitrypsin
ALT: alanine aminotransferase
anti‐HBc: antibodies against HBcAg
AP: alkaline phosphatase
AST: aspartate aminotransferase
BMI: body mass index
cART: combination antiretroviral therapy
CDT: carbohydrate‐deficient transferrin
CHE: cholinesterase
CLD: chronic liver disease
EASL: European Association for the Study of the Liver
GBD: Global burden of disease
GGT: gamma‐glutamyltransferase
GLDH: glutamate dehydrogenase
HBeAg: hepatitis B e antigen
HBsAg: hepatitis B surface antigen
HBV: hepatitis B virus
HCC: hepatocellular carcinoma
HCV: hepatitis C virus
HDV: hepatitis D virus
HEV: hepatitis E virus
HIV: human immunodeficiency virus
INR: international normalised ratio
LC: liver cirrhosis
LF: liver fibrosis
LMIC: low‐ and middle‐income countries
LSM: liver stiffness measurement
MASLD: metabolic dysfunction‐associated steatotic liver disease
metALD: metabolic dysfunction and alcohol‐related liver disease
MHBs: middle HBV surface protein
OBI: occult hepatitis B infection
OR: odds ratio
PLWH: people living with HIV
SSA: sub‐Saharan Africa
TE: transient elastography
US: ultrasound
WHO: World Health Organization

## 1 Introduction

Chronic liver diseases (CLD), liver cirrhosis (LC), and hepatocellular carcinoma (HCC) are highly prevalent in sub‐Saharan Africa (SSA) and are associated with high morbidity and mortality, especially in health care settings with limited diagnostic and therapeutic resources. For instance, liver disease was the third most common cause of death in a Nigerian study over a 14‐year period [1]. HCC is one of the most common cancers in SSA, but is usually diagnosed at late stages with a correspondingly short survival time [2, 3, 4, 5]. There is evidence that liver disease is particularly common in patients seeking medical care in SSA, but prevalence data are widely lacking. One study investigated liver disease patterns among elderly hospitalised patients in Rwanda and found LC to be present in 31.8% of patients and HCC in 5.7% of patients [6], while research from Uganda found fibrosis in 24% of participants based on the GGT‐to‐platelet‐ratio score [7].

Viral hepatitis, primarily caused by hepatitis B virus (HBV) and, to a lesser extent, hepatitis C virus (HCV), is also highly prevalent in SSA with national prevalence reaching up to 13.9% and 4.3%, respectively [8, 9]. There are an estimated 65 million HBV infections in the WHO African Region, where HBV is the leading or second‐leading cause of cirrhosis‐related deaths in Western, Eastern, Central and Southern Africa [9, 10]. Further, the prevalence of metabolic dysfunction‐associated steatotic liver disease (MASLD) in SSA ranges from 24.5% in the east to 34.3% in the west, with an average of 29%, which is somewhat lower compared to other regions [11]. However, the prevalence and importance of MASLD are growing worldwide and expected to become a central aspect for the clinical management of CLD [12].

The reliable diagnosis of liver fibrosis (LF) and LC in resource‐limited health care settings is challenging. Transient elastography (TE) has been established as a non‐invasive, rapid, and reproducible method for the diagnosis of LF and LC in lieu of ultrasound (US) examinations and has been evaluated in numerous studies worldwide for various aetiologies [13, 14].

As CLD and its underlying causes pose a significant burden on SSA, knowledge of the epidemiology and risk factors for CLD is essential for guiding screening and prevention strategies, and clinical management. Using TE as a reference standard, we conducted a cross‐sectional, hospital‐based study to assess the prevalence, aetiology and risk factors for LF/LC in patients attending one of Ghana's largest hospitals.

## 2 Methods

### 2.1 Patient Recruitment, Socioeconomic Data Survey and Initial Physical Examination

We recruited hospitalised patients and outpatients from the internal medicine department of the Komfo Anokye Teaching Hospital in Kumasi, Ghana. In July 2011, all patients > 18 years of age admitted to an internal medicine ward or presenting to the internal medicine outpatient clinics were offered inclusion in the study.

Written informed consent was obtained from all participants. Exclusion criteria included known pregnancy and advanced obesity. Medical and socioeconomic data were recorded with a standardised questionnaire. Medical history, clinical data, haemoglobin levels and platelet counts were extracted from medical records. Data are reported according to STROBE guidelines for cross‐sectional studies.

### 2.2 Transient Elastography

TE was conducted with a FibroScan 402 device (Echosens, France) using a standard probe. We considered the median value of ≥ 10 successful measurements valid if the success rate was > 60% and the interquartile range was < 30%. A significant LF (METAVIR score F ≥ 2) was defined as a liver stiffness measurement (LSM) ≥ 7.2 kPa, and a significant LC (METAVIR score F4) was defined as a LSM ≥ 11 kPa, as proposed by Marcellin et al. for patients with chronic hepatitis B, assuming this as the prevailing cause of liver disease in our setting [15].

### 2.3 Assessment of CLD Aetiology and Factors Interfering With TE

Investigations for the assessment of the aetiology of CLD in LF/LC patients and for factors interfering with TE were done in subgroups of patients according to liver status, stratified by LSM. We performed US of the abdomen, biochemical and serological analyses in all patients with an LSM ≥ 7.2 kPa (indicating fibrosis) and for an equally large subset of patients without LF/LC (LSM ≤ 6.0 kPa), serving as the control group. To maintain group balance, each patient with a pathological LSM was matched by allocating the subsequent patient with a normal LSM to blood sampling and ultrasonography.

For better discrimination, participants with LSM values between 6.0 and 7.2 kPa (termed ‘ambiguous’) as well as those with potential confounding factors affecting TE (such as liver tumours, ascites and acute hepatitis) were excluded from further analyses.

### 2.4 Ultrasound Examination

US was performed according to a standardised protocol using a Siemens Sonoline Adara (Siemens, Germany) and a Mindray 35C50EB (Bio‐Medical Electronics, China). In addition to detailed liver examination, a US scoring system for evaluating the fibrosis stage, which was adapted and simplified from Nishiura et al., was applied. The liver edge, surface and parenchymal texture were assessed and categorised as described [16]. The liver edge was scored 0 (sharp), 1 (mildly blunted) or 2 (blunted); the liver surface was scored as 0 (smooth), 1 (mildly irregular), or 2 (irregular). Parenchymal texture was scored as 0 (fine), 1 (mildly coarse) or 2 (coarse). US and TE were performed by experienced examiners who were blinded to other results and clinical information.

### 2.5 Virological Analyses

Serum and plasma samples were stored at −20°C and shipped to Germany. Qualitative measurements of anti‐HBc, HBsAg, HBeAg, anti‐HIV and anti‐HCV were performed on the Architect system (Abbott, Germany). In borderline cases, anti‐HCV was confirmed by immunoblotting (*recom*LINE HCV; Mikrogen Diagnostik, Germany). Anti‐HDV and anti‐HEV antibodies were detected via ELISA (HEV IgG EIA by Axiom, Germany and ETI‐AB_DELTAK‐2 by DiaSorin, Italy, respectively) following the manufacturer's instructions.

Viral nucleic acids were extracted using the QIAamp MinElute Virus Kit (Qiagen, Germany). The HBV load was determined by commercial real‐time PCR (RealStar HBV PCR kit 1.0; Altona Diagnostics, Germany). HBV genomic sequences were amplified with Platinum Taq DNA polymerase using previously described methods [17] and new primers (Table S1). Sanger sequencing was performed by LGC genomics (Germany). The HBV serotype was determined according to the methods of Purdy et al. [18], and genotyping was performed using Geno2Pheno[HBV] (https://hbv.geno2pheno.org/). The HCV load was determined by commercial real‐time RT‐PCR (RealStar HCV RT‐PCR Kit 1.0; Altona Diagnostics, Germany). For HCV RNA‐rpositive samples, nested RT‐PCR of an amplicon within the nonstructural protein 5b (NS5b) gene was performed as described [19], and genotyping was performed using Geno2Pheno[HCV] (https://hcv.geno2pheno.org/) [20]. HDV RNA was detected using in‐house real‐time RT‐PCR with previously published modified primers and probe [21, 22, 23]. HDV RNA was also amplified via conventional nested touchdown RT‐PCR using new primers located in the Delta antigen coding region designed through multiple sequence alignment of publicly available HDV sequences (Table S1) using the SuperScript III One‐Step RT‐PCR system (Invitrogen, Germany) and the following cycling program: 95°C for 3 min, 50 cycles of (94°C for 15 s, 60°C [1°C touchdown per cycle starting at cycle 11] for 20 s, 72°C for 30 s), 72°C for 2 min. The first round PCR was preceded by an RT step at 50°C for 20 min. The amplification product was Sanger sequenced and genotyped using the HDVdb [24]. HEV RNA was detected by commercial real‐time RT‐PCR (RealStar HEV RT‐PCR Kit 1.0; Altona Diagnostics, Germany).

### 2.6 Diagnosis of MASLD and metALD

We followed the diagnostic criteria recommended by the current guidelines of the European Association for the Study of the Liver (EASL) [25] to define MASLD as follows: Hepatic steatosis diagnosed in ultrasound combined with at least one cardiometabolic risk factor (i.e., arterial hypertension, diabetes mellitus, dyslipidemia) without concomitant chronic HBV infection or alcohol abuse (either anamnestic history of past or current alcohol abuse or elevated carbohydrate‐deficient transferrin [CDT]). Diagnosis of arterial hypertension according to anamnesis, diabetes according to anamnesis or by blood glucose > 200 mg/dL, dyslipidemia as serum triglycerides > 200 mg/dL and low HDL‐cholesterol as < 40 mg/dL. Metabolic dysfunction and alcohol‐related liver disease (metALD) diagnosis included the above‐mentioned requirements for MASLD with past or current heavy alcohol consumption. Body mass index (BMI) and waist circumference were not recorded.

### 2.7 Statistics

Univariate analyses were performed using GraphPad Prism (version 9.4.0). Continuous variables are expressed as the mean (±SD) or median (range) and were compared through Welch's *t*‐test. Fisher's exact test was applied for the comparison of categorical variables. *p* values less than 0.05 were considered statistically significant. The following tests were performed in the RStudio environment (version 2023.06.1): multivariate analysis via generalised, fixed effects, and logistic regression without interactions expressing the results as odds ratios (ORs) with 95% CIs, Nagelkerke's *R* ^2^ and Spearman's rank correlation coefficient (𝜌) to correlate CLD stage and US score. Missing data were treated under missing at random (MAR) assumptions.

## 3 Results

### 3.1 Patient Characteristics and Categorisation

**FIGURE 1:**
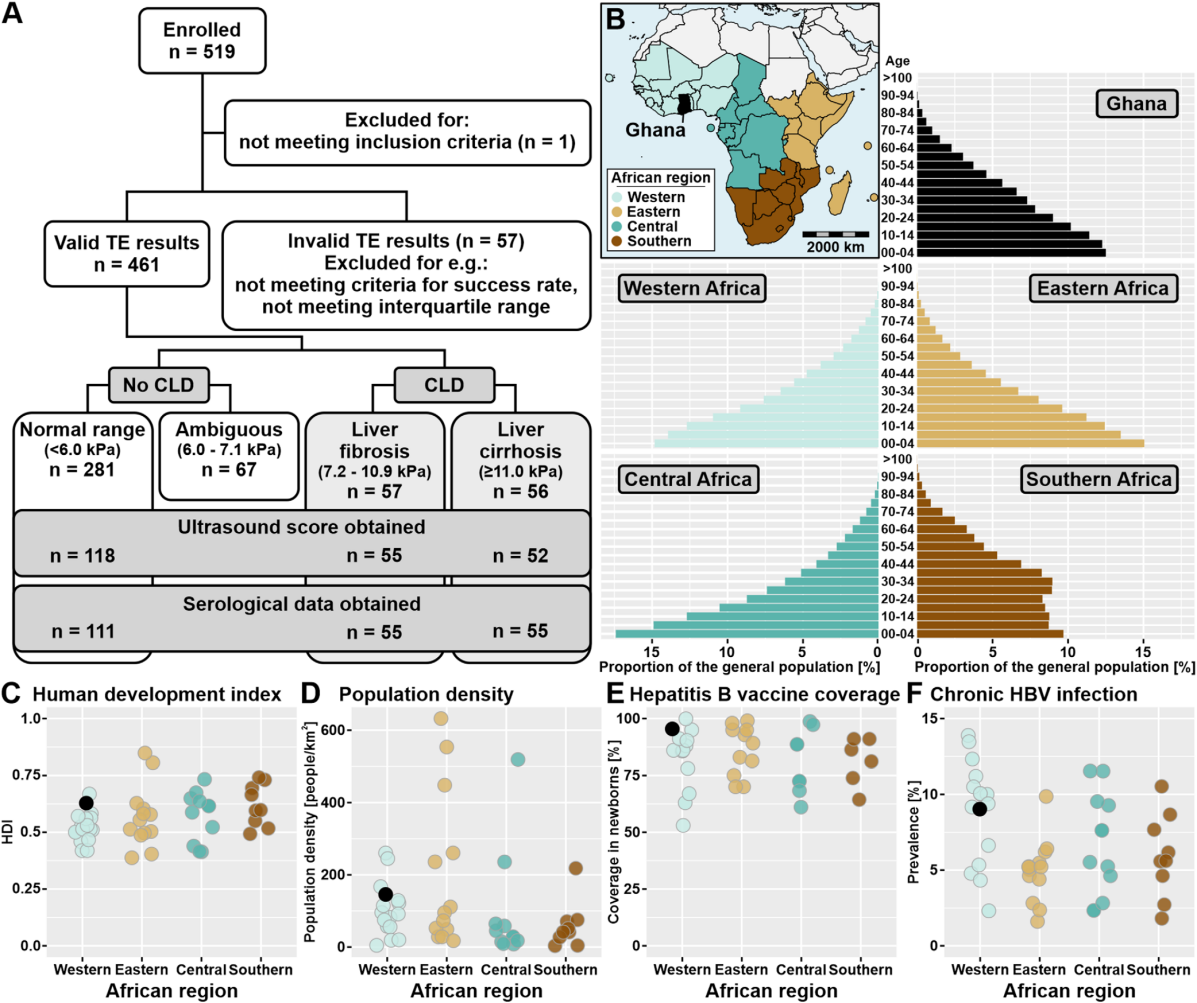
Study flow chart and demographic, developmental, and epidemiological parameters of sub‐Saharan African countries. (A) Study flow chart. (B) Colour‐coded map of the sub‐Saharan African geographical regions (as defined by the United Nations geoscheme) and their respective population distributions via the United Nations. Ghana, the focus of this study, is marked in black. Publicly available data from sub‐Saharan African countries on human development index via the United Nations Development Programme (C), population density via the World Bank (D), hepatitis B vaccine coverage in newborns via the World Health Organization (E), and prevalence of chronic HBV infection via the World Health Organization (F). Map of Africa created based on freely available data implemented in the R package ‘maps’ v3.4.2.1.

We recruited a total of 519 patients in Kumasi, Ghana (Figure 1A), as Ghana can be considered reflective of large parts of SSA in terms of age demographics, human development, population density, and HBV prevalence (Figure 1B–F). One patient was excluded due to a protocol violation (underage). Valid liver stiffness measurements (LSMs) were obtained for 461 patients, of whom 198 (43.0%) were male, 100 (21.7%) were hospitalised, and the mean age was 48.3 (±15.1) years (Table 1). The majority were Christian (85.8%, 392/457), 20.6% (95/461) had no formal education, 23.3% (107/460) were currently unemployed, and 9.1% (42/461) reported past or current alcohol abuse, defined as consumption of 100 mL or more of ethanol weekly.

**TABLE 1:** Study cohort characteristics grouped by liver stiffness measurement (LSM) categories. Continuous variables were compared through two‐tailed *t*‐tests with Welch's correction. Fisher's exact test was applied for the comparison of categorical variables. *p value*s less than 0.05 were considered statistically significant.

Characteristics	Total, *n* = 394	Control (LSM ≤ 6.0 kPa), *n* = 281	Liver fibrosis (LSM 7.2–10.9 kPa), *n* = 57		Liver cirrhosis (LSM ≥ 11.0 kPa), *n* = 56	
	Mean (±SD) or *n* (%)	Mean (±SD) or *n* (%)	Mean (±SD) or *n* (%)	*p value* compared to control	Mean (±SD) or *n* (%)	*p value* compared to control
Age	48.2 (±15.4)	48.1 (±15.6)	48.4 (±13.3)	0.899	48.5 (±16.5)	0.879
Male sex	172 (43.7)	103 (36.7)	32 (56.1)	**0.008**	37 (66.1)	**< 0.001**
Hospitalisation	92 (23.4)	47 (16.7)	17 (29.8)	**0.027**	28 (50.0)	**< 0.001**
Christian faith	336 (86.2)	245 (88.1)	45 (79.0)	0.143	46 (83.6)	0.295
Current unemployment	98 (24.9)	68 (24.2)	13 (22.8)	> 0.999	17 (30.9)	0.399
No formal education	77 (19.5)	47 (16.7)	14 (24.6)	0.186	16 (28.6)	0.059
Tap water in the household	262 (66.7)	180 (64.3)	38 (66.7)	0.763	44 (78.6)	**0.043**
Electricity in the household	359 (91.6)	256 (91.8)	50 (87.7)	0.456	53 (94.6)	0.595
Self‐reported alcohol abuse (past or present)	39 (9.9)	19 (6.8)	10 (17.5)	**0.016**	10 (17.9)	**0.015**
Regular intake of herbal preparations	70 (17.8)	41 (14.6)	14 (24.6)	0.076	15 (26.8)	**0.031**
Known HIV infection	78 (20.0)	67 (23.9)	6 (10.7)	**0.033**	5 (9.1)	**0.012**
Known HBV infection	45 (11.5)	26 (9.3)	7 (12.7)	0.467	12 (21.8)	**0.018**
Known liver disease	25 (6.5)	9 (3.3)	2 (3.5)	> 0.999	14 (26.4)	**< 0.001**
Known diabetes mellitus	157 (40.1)	114 (40.6)	28 (50.0)	0.236	15 (27.3)	0.070
Known hypertension	132 (33.6)	100 (35.6)	19 (33.3)	> 0.999	23 (23.6)	0.294
History of jaundice	59 (15.0)	32 (11.4)	9 (15.8)	0.374	18 (32.1)	**< 0.001**
History of ascites	12 (3.1)	2 (0.7)	2 (3.5)	0.134	8 (14.6)	**< 0.001**

*Note:* *p* values below 0.05 are shown in bold to highlight that there is a significant difference between two compared groups.

Abbreviations: HBV: hepatitis B virus, HIV: human immunodeficiency virus, SD: standard deviation.

LSMs were pathological (≥ 7.2 kPa) in 24.5% (113/461) patients, of which 12.4% (57/461) and 12.1% (56/461) had LSMs indicative of LF (7.2–10.9 kPa) and LC (≥ 11.0 kPa), respectively. Blood sampling for virological and biochemical analysis and ultrasound examination was performed on 55 patients with LF and LC each and a similarly sized group of patients with normal LSM (≤ 6.0 kPa).

### 3.2 Risk Factors for CLD According to Univariate and Multivariate Analyses

Patient characteristics grouped by TE‐defined liver status are summarised in Table 1. Compared to individuals with normal LSM, those with advanced liver disease (LF + LC) were more often male (61.1% [69/113] vs. 36.7% [103/281], *p* < 0.001), hospitalised (39.8% [45/113] vs. 16.7% [47/281], *p* < 0.001), and reported current or previous alcohol abuse (17.7% [20/113] vs. 6.8% [19/281], *p* = 0.02). Only 3.5% (2/57) of patients with LF and 26.4% (14/53) of patients with LC reported prior knowledge of their liver disease. The overall prevalence of CLD according to these selected risk factors is shown in Figure S1.

To expand on this, we performed multivariate logistic regression analysis (Table 2) of the risk factors that were significantly associated with LF/LC in the univariate analysis. None of the tested parameters were significantly associated with the presence of LF. For LC, on the other hand, known HBV infection (OR: 9.2, 95% CI: 3.1–27.6), hospitalisation (3.0, 1.3–7.1), male sex (2.8, 1.2–6.4), lacking formal education (4.1, 1.7–9.8), and self‐reported history of ascites (12.8, 2.0–82.5) were confirmed as risk factors. Known HIV infection was inversely associated with the risk of LC (0.2, 0.1–0.9).

**TABLE 2:** Multivariate analysis of risk factors for liver fibrosis and liver cirrhosis. Multivariate analysis via generalised, fixed effects, and logistic regression without interactions expressing the results as odds ratios (ORs) with 95% CI was performed within the R Studio environment. *p value*s less than 0.05 were considered statistically significant.

Characteristics	Liver fibrosis			Liver cirrhosis		
	OR	95% CI	*p*	OR	95% CI	*p*
Male sex	1.795	0.903–3.568	0.095	2.821	1.240–6.420	**0.013**
Hospitalisation	1.196	0.511–2.800	0.680	3.008	1.276–7.091	**0.012**
No formal education	1.974	0.923–4.221	0.079	4.079	1.697–9.804	**0.002**
Tap water in the household	1.056	0.555–2.011	0.867	2.152	0.942–4.919	0.069
Regular intake of herbal preparations	1.235	0.563–2.710	0.599	1.120	0.476–2.635	0.795
Self‐reported alcohol abuse (past or present)	1.982	0.786–4.996	0.147	1.733	0.602–4.986	0.308
Known HIV infection	0.375	0.137–1.028	0.057	0.247	0.067–0.910	**0.036**
Known HBV infection	2.581	0.926–7.191	0.070	9.213	3.075–27.608	**< 0.001**
History of jaundice	0.945	0.362–2.463	0.907	1.428	0.575–3.549	0.442
History of ascites	3.463	0.271–44.181	0.339	12.751	1.971–82.486	**0.008**

*Note:* Liver fibrosis: *n* = 330, Nagelkerke's *R* ^2^ = 0.107; liver cirrhosis: *n* = 328, Nagelkerke's *R* ^2^ = 0.348. *p* values below 0.05 are shown in bold to highlight that there is a significant difference between two compared groups.

Abbreviations: CI: confidence interval, HBV: hepatitis B virus, HIV: human immunodeficiency virus, OR: odds ratio.

### 3.3 Biochemical Parameters

The biochemical and virological data grouped by TE‐defined liver status are shown in Table 3. Compared to patients in the control group, patients in the LC group had significantly greater concentrations of serum transaminases (alanine aminotransferase [ALT] 56.3 ± 118.6 vs. 19.3 ± 13.6 U/L, *p* = 0.025; aspartate aminotransferase [AST] 132.4 ± 188.8 vs. 29.0 ± 24.9 U/L, *p* < 0.001) and several other liver‐related biochemical parameters. Further, serum triglycerides and HDL‐cholesterol were significantly increased (148.84 ± 86.91 vs. 118.33 ± 50.42, *p* = 0.024) and decreased (36.27 ± 13.57 vs. 45.21 ± 16.66, *p* = 0.001) in LC patients compared to controls, respectively. In LF patients, only the serum levels of glutamate dehydrogenase were significantly elevated (7.0 ± 10.9 vs. 3.7 ± 3.4 U/L, *p* = 0.031). Alpha‐1 antitrypsin was within the normal range for all patients. Elevated transferrin saturation was found in one patient without liver disease (0.9%, 1/111), 3 patients (5.5%, 3/55; *p* = 0.107) with LF and 5 patients (9.1%, 5/55; *p* = 0.016) with LC. Carbohydrate‐deficient transferrin (CDT) levels were elevated in 9.8% (9/92), 12.5% (6/48, *p* = 0.774) and 15.2% (7/46, *p* = 0.401) of control, LF and LC patients, respectively. Of the 22 patients with elevated CDT levels, only 2 (9.1%) reported current alcohol abuse.

**TABLE 3:** Biochemical and virological parameters of the study cohort. Continuous variables were compared through two‐tailed *t*‐tests with Welch's correction. Fisher's exact test was applied for the comparison of categorical variables. *p value*s less than 0.05 were considered statistically significant.

	Controls *n* = 111	Liver fibrosis *n* = 55		Liver cirrhosis *n* = 55	
	Mean (±SD) or *n* (%)	Mean (±SD) or *n* (%)	*p value* compared to control	Mean (±SD) or *n* (%)	*p value* compared to control
Biochemical parameters					
AST (U/L)	29.0 (±24.9)	50.4 (±80.4)	0.059	132.4 (±188.8)	**< 0.001**
ALT (U/L)	19.3 (±13.6)	26.3 (±41.9)	0.233	56.3 (±118.6)	**0.025**
GGT (U/L)	57.6 (±55.7)	85.9 (±116.3)	0.092	274.9 (±341.3)	**< 0.001**
GLDH (U/L)	3.7 (±3.4)	7.0 (±10.9)	**0.031**	18.7 (±59.6)	0.069
AP (U/L)	86.1 (±40.3)	102.2 (±83.6)	0.185	211.3 (±315.7)	**0.006**
Bilirubin (mg/L)	0.7 (±1.6)	0.6 (±0.6)	0.691	2.4 (±4.5)	**0.008**
Albumin (g/L)	40.9 (±5.1)	39.0 (±7.7)	0.108	34.2 (±8.5)	**< 0.001**
CHE (IU/ml)	7.5 (±2.5)	7.3 (±3.3)	0.574	4.8 (±3.3)	**< 0.001**
INR	1.1 (±0.2)	1.1 (±0.2)	0.960	1.5 (±0.7)	**< 0.001**
Ferritin (ng/mL)	251.3 (±333.0)	434.8 (±1011.1)	0.199	473.2 (±582.8)	**0.012**
A1AT decreased	0 (0.0)	0 (0.0)	n.a.	0 (0.0)	n.a.
Transferrin saturation > 65%	1 (0.9)	3 (5.5)	0.107	5 (9.1)	**0.016**
CDT elevated (> 2.5%)	9 (9.8)	6 (12.5)	0.774	7 (15.2)	0.401
Total cholesterol (mg/dL)	170.28 (±43.5)	177.31 (±102.77)	0.631	155.74 (±78.33)	0.2239
HDL‐cholesterol (mg/dL)	45.21 (±16.66)	40.98 (±17.49)	0.148	36.27 (±13.57)	**0.001**
LDL‐cholesterol (mg/dL)	93.65 (±33.88)	114.0 (±139.04)	0.294	77.84 (±60.64)	0.089
Triglycerides (mg/dL)	118.33 (±50.42)	132.54 (±59.97)	0.137	148.84 (±86.91)	**0.024**
Serum glucose (mg/dL)	125.14 (±90.87)	151.19 (±103.3)	0.117	120.26 (±74.18)	0.714
Virological parameters					
Anti‐HIV	30 (27.0)	9 (16.4)	0.173	6 (10.9)	**0.017**
Anti‐HBc	89 (80.2)	49 (89.1)	0.189	52 (94.6)	**0.020**
HBsAg	14 (15.7)	7 (14.3)	> 0.999	20 (38.5)	**< 0.001**
HBeAg	1 (1.1)	1 (2.0)	> 0.999	10 (19.2)	**< 0.001**
HBV DNA	19 (17.1)	11 (20.0)	0.672	30 (54.6)	**< 0.001**
HBV DNA titre (log_10_ IU/mL)	2.0 (±1.9)	3.4 (±2.3)	0.114	4.6 (±2.9)	**< 0.001**
OBI	6 (6.7)	5 (10.2)	0.520	9 (17.3)	0.086
Anti‐HDV	2 (1.8)	2 (3.8)	0.596	4 (7.3)	0.096
HDV‐RNA	0 (0.0)	0 (0.0)	n.a.	1 (1.8)	0.331
Anti‐HCV	4 (3.6)	5 (9.1)	0.159	6 (10.9)	0.084
HCV RNA	0 (0.0)	1 (1.8)	0.331	2 (3.6)	0.108
HCV RNA titre (log_10_ IU/mL)	n.a.	5.0 (n.a.)	n.a.	5.0 (±0.8)	n.a.
Anti‐HEV	32 (28.8)	14 (25.5)	0.715	23 (41.8)	0.115
HEV RNA	0 (0.0)	0 (0.0)	n.a.	0 (0.0)	n.a.

*Note:* *p* values below 0.05 are shown in bold to highlight that there is a significant difference between two compared groups.

Abbreviations: A1AT, alpha‐1 antitrypsin; ALT, alanine aminotransferase; AP, alkaline phosphatase; AST, aspartate aminotransferase; CDT, carbohydrate‐deficient transferrin; CHE, cholinesterase; GGT, gamma‐glutamyltransferase; GLDH, glutamate dehydrogenase; HBV, hepatitis B virus; HCV, hepatitis C virus; HDL, high density lipoprotein; HDV, hepatitis D virus; HEV, hepatitis E virus; INR, international normalised ratio; LDL, low density lipoprotein; n.a., not applicable; OBI, occult hepatitis B infection; SD, standard deviation.

### 3.4 Virological Parameters

We detected HBV DNA in 27.1% (60/221) of patients, corresponding to active viral replication. HBV DNA was found more frequently and at higher viral loads in patients with LC than in patients with normal LSM (54.5% [30/55] vs. 17.1% [19/111], *p* < 0.001 and 4.6 ± 2.9 vs. 2.0 ± 1.9 log_10_ IU/ml, *p* < 0.001, respectively). We also found a high overall prevalence of anti‐hepatitis B core antibodies (anti‐HBc), indicating current or previous infection with HBV (86.0%, 190/221) (Table 3). The anti‐HBc prevalence was significantly higher in LC patients than controls (94.6% [52/55] vs. 80.2% [89/111], *p* = 0.020). Of those with anti‐HBc, 21.6% (41/190) were positive for HBsAg, of which nearly all had detectable HBV DNA levels (92.7%, 38/41). Among those who were negative for HBsAg, 13.4% (20/149) had HBV DNA indicating occult HBV infection (OBI) [26]. In two cases, HBV DNA was detected despite anti‐HBc‐seronegativity. HBeAg was detectable in 12 HBV‐DNA‐positive patients (20.0%, 12/60), who had significantly higher AST levels (257.5 ± 294.6 vs. 51.4 ± 71.7 U/L, *p* = 0.041) and LSM values (41.1 ± 27.7 vs. 18.4 ± 20.8 kPa, *p* = 0.023) compared to HBeAg‐negative participants. Antibodies against HDV were detected in eight patients among those with detectable HBV DNA (13.3%, 8/60), with one patient also being HDV RNA positive (1.7%, 1/60).

Anti‐HCV was positive in 6.8% (15/221) of patients overall, but HCV RNA was detected in only one patient with LF (1.8%, 1/55) and two patients with LC (3.6%, 2/55). Anti‐HEV antibodies were detected in 28.8% (32/111) of the controls, 25.5% (14/55) of the LF patients, and 41.8% (23/55; *p* = 0.115) of the LC patients. Anti‐HIV antibodies were detected in 27.0% (30/111) of the control patients, 16.4% (9/55; *p* = 0.173) of the LF patients, and 10.9% (6/55; *p* = 0.017) of the LC patients.

### 3.5 Molecular Analysis of Hepatitis Viruses

Thirty‐six partial to full HBV genomes were obtained through multi‐amplicon nested PCR (Table S2; GenBank acc. no. PX716963‐PX716998). All recovered sequences belonged to HBV genotype E, apart from one sample of subgenotype A1 (LSG‐518). Antiviral drug resistance and immune escape mutations were found in 16.7% (6/36) and 36.1% (13/36) of the isolates, respectively. The latter are of special interest, as mutations in the preS/S region have been linked to accelerated liver disease progression [27, 28, 29]. Mutations affecting the expression of the immunomodulatory HBeAg were detected in all of the isolates for which the corresponding region (basal core promoter/preC) could be amplified. The majority of available sequences (7/13; 53.8%) showed preS2 start codon mutations abrogating middle surface protein (MHBs) expression or had other mutations (e.g., deletions) indicative of prolonged HBV infection [30, 31].

Nested PCR of the single HDV‐RNA positive sample revealed infection with genotype 1 (GenBank acc. no. PX716962) and sequencing of an NS5b amplicon of the three HCV‐RNA‐positive samples indicated infection with genotype 2 in all (GenBank acc. no. PX716959‐PX716961).

### 3.6 Aetiology of CLD in Fibrosis/Cirrhosis Patients

**FIGURE 2:**
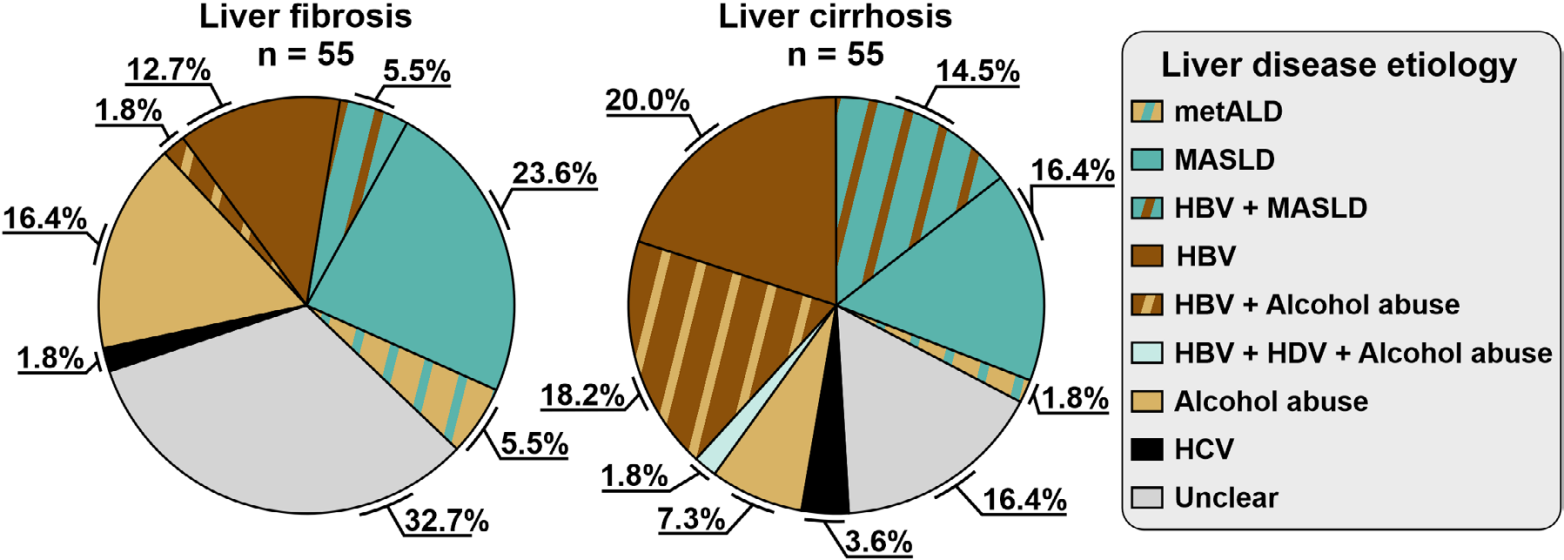
Putative aetiologies of liver fibrosis and liver cirrhosis in the study cohort. Aetiologies were defined as: active hepatitis B (HBV DNA positivity), alcohol abuse (self‐reported current or past alcohol abuse and/or elevated carbohydrate‐deficient transferrin level), active hepatitis C (HCV RNA positivity), active hepatitis D (HDV RNA positivity), MASLD (hepatic steatosis with anamnestic hypertension, anamnestic diabetes mellitus or serum glucose > 200 mg/dL, serum triglycerides > 200 mg/dL or HDL‐cholesterol < 40 mg/dL, without HBV infection or alcohol abuse), metALD (MASLD with concomitant alcohol abuse) or unclear (no apparent indicator).

We assigned six possible underlying causes of CLD (present or past alcohol abuse, MASLD, metALD, HBV, HCV, and/or HDV infection) that represented 83.6% (46/55) and 67.3% (37/55) of LC and LF cases, respectively (Figure 2). We detected active HBV infection in 54.5% (30/55) of participants with LC, of which 36.7% (11/30) also showed evidence of high alcohol consumption (either through self‐reporting or via elevated CDT). We identified MASLD and metALD in 16.4% (9/55) and 1.8% (1/55) of LC patients, respectively. In 7.3% (4/55), alcohol consumption was the only evident cause of LC. In participants with LF, MASLD (23.6%, 13/55) and heavy alcohol consumption (23.6%, 13/55) were the predominant underlying causes. Of the latter, three (23.1%, 3/13) had metALD and one (7.7%, 1/13) had concomitant HBV infection. Active HBV infection was found in 20% (11/55). HCV and HDV were of minor importance for CLD (LF: 1.8% [1/55] and 0%; LC: 3.6% [2/55] and 1.8% [1/55], respectively). No patient showed signs of alpha‐1‐antitrypsin deficiency. We found indication of iron overload (defined as transferrin saturation > 65% and ferritin > 300 ng/mL) was found in five patients with LC and three with LF. There was no genetic testing, magnetic resonance imaging and/or histology available to confirm hemochromatosis/iron overload.

## 4 Discussion

Here, we report a high prevalence of liver disease (LF/LC) in patients who presented to the internal medicine services of a university hospital in Ghana. Using TE, LF/LC was diagnosed in almost a quarter (24.5%) of patients, with liver fibrosis (LF) being present in 12.4% and liver cirrhosis (LC) in 12.1% of patients. Hospitalisation, male sex, current or past alcohol abuse, lack of formal education, and known HBV infection were risk factors for CLD. Remarkably, LC was found in 28% of hospitalised patients, with only a quarter of patients being aware of the pathology and the associated higher risk for complications and mortality. The findings of our study suggest a major burden of disease associated with CLD for the health systems in Ghana. Despite the scarcity of data related to CLD prevalence in SSA, we consider our findings to be likely transferable to other parts of SSA, as Ghana is representative of many African countries in regard to age distribution, human development index, and population density (Figure 1B–D). This is substantiated by a study from Rwanda reporting a similar prevalence of LC of 31.8% among hospitalised patients [6]. The findings suggest that hospitals may provide important screening opportunities for CLD and that diagnostic workup for patients presenting to internal medicine should include liver pathologies, given the broad range of associated complications and its implications for the prognosis. A basic diagnostic panel may identify an underlying and treatable cause in 75% of patients, with the perspective of preventing or delaying progress of the disease and thereby significantly improving the prognosis of those affected.

HBV infection was the predominant risk factor for CLD in this study, with active HBV infection being present in 54.5% of patients with LC, 20% in patients with LF, and 17.1% in patients without detectable liver disease. Our data are in accordance with a cross‐sectional Ethiopian study reporting 36.7% of CLD attributable to chronic hepatitis B infection [32] and likely generalizable to other countries in SSA with comparable prevalence of chronic hepatitis B infection (Figure 1F).

Remarkably, the frequency of OBI (HBV DNA positivity despite HBsAg negativity) was high, reaching 16.7% in patients with LC in our study. A recent investigation on people living with HIV (PLWH) in Ghana found a similarly high prevalence of OBI [33]. Taken together, these data suggest that current HBV screening strategies based on HBsAg testing alone may be inadequate. As the high burden of HBV in sub‐Saharan Africa is increasingly recognised, new treatment programs are established and screening activities are becoming increasingly important. According to the EASL and WHO guidelines for the treatment of HBV, antiviral treatment was indicated in 24 out of 60 (40%) patients with active hepatitis B, translating to a number‐needed‐to‐screen of 19.2.

The prevalence of active HCV infection was low in our cohort (1.4%) with regard to published data ranging from < 1% to 9.4% [34, 35, 36, 37]. A study conducted by Blankson and colleagues, who investigated the role of HBV and HCV in 70 patients with LC in Accra, Ghana, revealed that 43% were HBsAg‐ and 7% anti‐HCV‐positive [38]. HEV, for which sporadic cases of chronic infections have been described, had no role in CLD in this study population as we did not detect HEV RNA in any of the participants.

In addition to viral hepatitis, MASLD and alcohol consumption, as non‐communicable diseases, contributed substantially to disease burden in this cohort, either individually or as co‐factors. MASLD was present as the sole apparent aetiology in a higher proportion of LF compared to LC patients (23.6% [13/55] and 16.4% [9/55]). Including patients with MASLD and HBV, a similar proportion of patients met MASLD definitions in LF and LC patients (29.1% [16/55] vs. 30.9% [17/55]), in line with previously reported data [11]. There was evidence of recent or past heavy alcohol consumption in almost one third (29.1%, 16/55) of patients with LC. Our data also suggest alcohol to play an important role as a co‐factor, as more than two thirds of participants with high alcohol consumption were also infected with HBV. This is in line with a meta‐analysis based in Ethiopia revealing viral hepatitis and alcohol misuse as frequent etiologies (40% and 17% pooled estimates, respectively) [39]. Since only 9.1% (2/22) of patients with elevated CDT levels reported current alcohol consumption, data on self‐reported alcoholism should be interpreted with caution in future studies.

Notably, the prevalence of LF/LC was lower in HIV‐positive compared to HIV‐negative patients (33.3% [15/45] vs. 54.0% [95/176], *p* = 0.019), most likely reflecting the close medical care offered for PLWH, including screening for HBV in most settings, as well as the efficacy of combination antiretroviral therapy (cART) on HBV infection. However, HIV infection has been linked to CLD [40] and may accelerate the disease progression of, for example, chronic hepatitis C [41]. A recent study among PLWH in Uganda reported a reduction of fibrosis risk by cART, but in contrast to our study a higher prevalence in HIV‐positive compared to HIV‐negative individuals (17% vs. 11%) [42].

Our results further suggest the suitability of TE as a non‐invasive point‐of‐care screening tool for LF/LC in high‐prevalence and low‐resource health care settings, where, in the absence of clear stigmata of LC, the diagnosis of LF and LC is difficult to establish with medical history and clinical examination providing the most important guidance for the suspicion of CLD. Our data show a moderately positive overall correlation of an established US score with LSM (Spearman's rank correlation coefficient 𝜌 = 0.56 [*p* < 0.001]) (Figure S2).

Although our study contributes important data on prevalence and spectrum of liver diseases among patients presenting for medical care in SSA, several limitations need to be addressed. The samples presented here were collected in 2011 and may not be fully representative of the current situation. However, population‐scale data by the Global Burden of Disease (GBD) consortium [43, 44] shows an increase in the rate of HBV‐related deaths in Ghana between 2011 and 2021 (Figure S3). From a long‐term public health perspective, universal hepatitis B vaccination has the potential to substantially reduce the burden of liver disease linked to the infection. However, since implementation of a vaccination program against hepatitis B in children was only initiated in 2002 in Ghana, HBV is likely to remain a dominant factor in CLD for the next decades in Ghana and other countries in SSA with similarly high levels of vaccination coverage in newborns (Figure 1E). Lastly, since we collected serum samples, determination of recent heavy alcohol exposure by biomarkers with superior sensitivity compared to CDT (i.e., phosphatidylethanol) using whole blood could not be performed, limiting conclusions drawn on alcohol‐driven CLD.

Importantly, TE is only a proxy for histopathological changes, with biopsy representing the gold standard, as several factors (e.g., acute hepatitis, obstructive jaundice, vascular congestion of the liver and liver tumours) may interfere with LSM results [45, 46, 47, 48]. Although we performed biochemical analyses and US in order to exclude patients with those conditions, undetected cases might still have led to an overestimation of LF/LC in our study population. On the other hand, the per‐protocol exclusion of patients with ascites and hepatic encephalopathy likely caused an underestimation of the prevalence of CLD in our study population, although most of these represent obvious cases of liver cirrhosis and are unlikely to be missed even without transient elastography. As we assumed HBV to be the prevailing cause of CLD in the study region, the LSM cut‐off values used to define significant LF (≥ 7.2 kPa) and LC (≥ 11 kPa) were adopted from a study among patients with HBV in France [15]. In this regard, a meta‐analysis by Friedrich‐Rust et al. reported optimal cut‐off values of 7.65 and 13.01 kPa for significant LF and LC, respectively [14]. The use of those cut‐offs would result in a slightly lower prevalence of CLD in our study population (20.8% [96/461] vs. 24.5% [113/461], *p* = 0.208), without relevant impact on our study findings and conclusions. Likewise, the biochemical parameters are only approximations and prone to interference: Ferritin and transferrin saturation, as indicators of iron overload, can also be elevated in infections or during inflammation, and CDT, a reliable indicator for high alcohol consumption, can be influenced by primary biliary cholangitis, HCC, HCV and high BMI [49]. Furthermore, we did not investigate other liver diseases, such as autoimmune hepatitis and primary sclerosing cholangitis. Because no data on waist circumference or BMI was available, the impact of MASLD cannot be determined, but should be included in future studies.

Finally, since we included only persons presenting for medical care, results may not be representative of the general population. Since resources in SSA are limited, presenting at medical care seems to be a good opportunity for CLD screening via TE, but may miss CLD before progression to LF/LC. In Europe, screening of the general population is currently being studied [50] and may offer the chance to detect more CLD before the development of LF/LC.

## 5 Conclusion

Our findings demonstrate a high burden of CLD among people seeking medical care in a large Ghanaian hospital, with HBV, MASLD and alcohol‐associated liver disease being the predominant risk factors. Despite viral hepatitis remaining the predominant single cause of CLD, non‐communicable diseases comprise important independent co‐factors and should be integrated into clinical care pathways and prevention strategies. Our data corroborate recent reports on viral hepatitis, with liver cirrhosis and HCC as the most important complications, being highly prevalent and among the most pressing public health concerns in SSA [51]. Our findings further underline the urgency of suitable screening, prevention, and management programs for viral hepatitis and CLD and demonstrate that health care institutions may represent ideal screening portals. Nevertheless, more current data on the prevalence and spectrum of CLD, also beyond factors investigated in this study, across SSA are needed to guide appropriate treatment and prevention measures for this highly affected region. We propose a screening with TE and, in cases with pathological results, further targeted laboratory and US diagnostic workup, the latter particularly for HCC screening in this high‐risk group. In patients with established LC, regular surveillance for HCC is warranted. The short examination time, the relatively low training requirements, and the low maintenance costs make TE a suitable screening tool, but the high costs of purchase limit the availability in SSA and other low‐ and middle‐income countries (LMIC). Privileged purchase mechanisms for LMIC could improve the availability of TE. The knowledge of risk factors, as described in our study, also allows implementation of targeted screening initiatives. In any case, the capacity for the prevention, diagnosis, and management of complications of CLD needs to be scaled up significantly in SSA. Systematic surveillance of the burden of disease and associated risk factors is needed in order to guide the implementation of such programs.

## Author Contributions

**Felix Lehmann:** formal analysis, investigation, data curation, writing – original draft, writing – review and editing, validation, visualisation; **Alexander Killer:** formal analysis, data curation, writing – original draft, writing – review and editing, validation; **Sarah Wels:** formal analysis, investigation, data curation, validation; **Stefan Schmiedel:** conceptualisation, investigation, supervision, writing – review and editing; **Richard Odame Phillips:** conceptualisation, investigation, supervision, writing – review and editing; **Pia Luise Roppert:** formal analysis, investigation, data curation, methodology; **Kirsten Alexandra Eberhardt:** resources, writing – original draft, writing – review and editing; **Martha Charlotte Holtfreter:** investigation, formal analysis, data curation, validation; **Sabine Stauga:** investigation, supervision, writing – original draft; **Ansgar Wilhelm Lohse:** conceptualisation, writing – original draft, writing – review and editing; **Stephan Ehrhardt:** formal analysis, data curation, writing – original draft, writing – review and editing; **Ohene Opare‐Sem:** conceptualisation, investigation, supervision, writing – original draft, writing – review and editing; **Hans Martin Orth:** writing – review and editing, supervision; **Fred Stephen Sarfo:** conceptualisation, investigation, supervision, writing – original draft, writing – review and editing; **Christian Drosten:** resources, supervision; **Anna Maria Eis‐Hübinger:** formal analysis, data curation, methodology, writing – original draft; **Tom Luedde:** writing – review and editing, supervision; **Dieter Glebe:** resources, formal analysis, writing – review and editing, funding acquisition; **Jan Felix Drexler:** conceptualisation, resources, writing – original draft, writing – review and editing, supervision, project administration, funding acquisition; **Torsten Feldt:** conceptualisation, resources, writing – original draft, writing – review and editing, supervision, project administration, funding acquisition.

## Funding

This research was supported by the Deutsche Forschungsgemeinschaft (DFG) project number 537500489 (Jan Felix Drexler and Dieter Glebe) and by the YAEL Foundation for Liver Research and Medical Education. The National Reference Centre for Hepatitis B Viruses and Hepatitis D Viruses at Justus Liebig University Giessen (Dieter Glebe) is supported by the German Ministry of Health via the Robert Koch Institute, Berlin, Germany.

## Ethics Statement

This research was approved by the ethics committee of the Kwame Nkrumah University of Science and Technology that oversees the Komfo Anokye Teaching Hospital (ref. CHRPE/55/11).

## Conflicts of Interest

The authors declare no conflicts of interest.

## Supporting information


**Data S1:** liv70538‐sup‐0001‐Supinfo.docx.

## Data Availability

All original data are made available in anonymised form through Jan Felix Drexler or Torsten Feldt upon reasonable request. Consensus sequences of HBV, HCV and HDV are accessible under GenBank acc. no. PX716959‐PX716998.
