# Supplementary material for: Prevalence and Spectrum of Chronic Liver Disease Among Patients Seeking Health Care in Ghana
Source: Liver Int. 2026 Feb 14;46(3):e70538. doi: 10.1111/liv.70538 (PMC12905631; doi:10.1111/liv.70538)
Supplement: Supplementary file 1 — Data S1: liv70538‐sup‐0001‐Supinfo.docx. [file LIV-46-0-s001.docx]

Supplementary material for

**Prevalence and spectrum of chronic liver disease among patients seeking health care in Ghana**

Felix Lehmann*, Alexander Killer*, Sarah Wels, Stefan Schmiedel, Richard Odame Phillips, Pia Luise Roppert, Kirsten Alexandra Eberhardt, Martha Charlotte Holtfreter, Sabine Stauga, Ansgar Wilhelm Lohse, Stephan Ehrhardt, Ohene Opare-Sem, Hans Martin Orth, Fred Stephen Sarfo, Christian Drosten, Anna Maria Eis-Hübinger, Tom Luedde, Dieter Glebe, Jan Felix Drexler^#^ and Torsten Feldt^#^

*,# Authors contributed equally

**This supplementary file contains:**

Figures S1-3

Tables S1-2

Supplementary references

**SUPPLEMENTARY FIGURES**


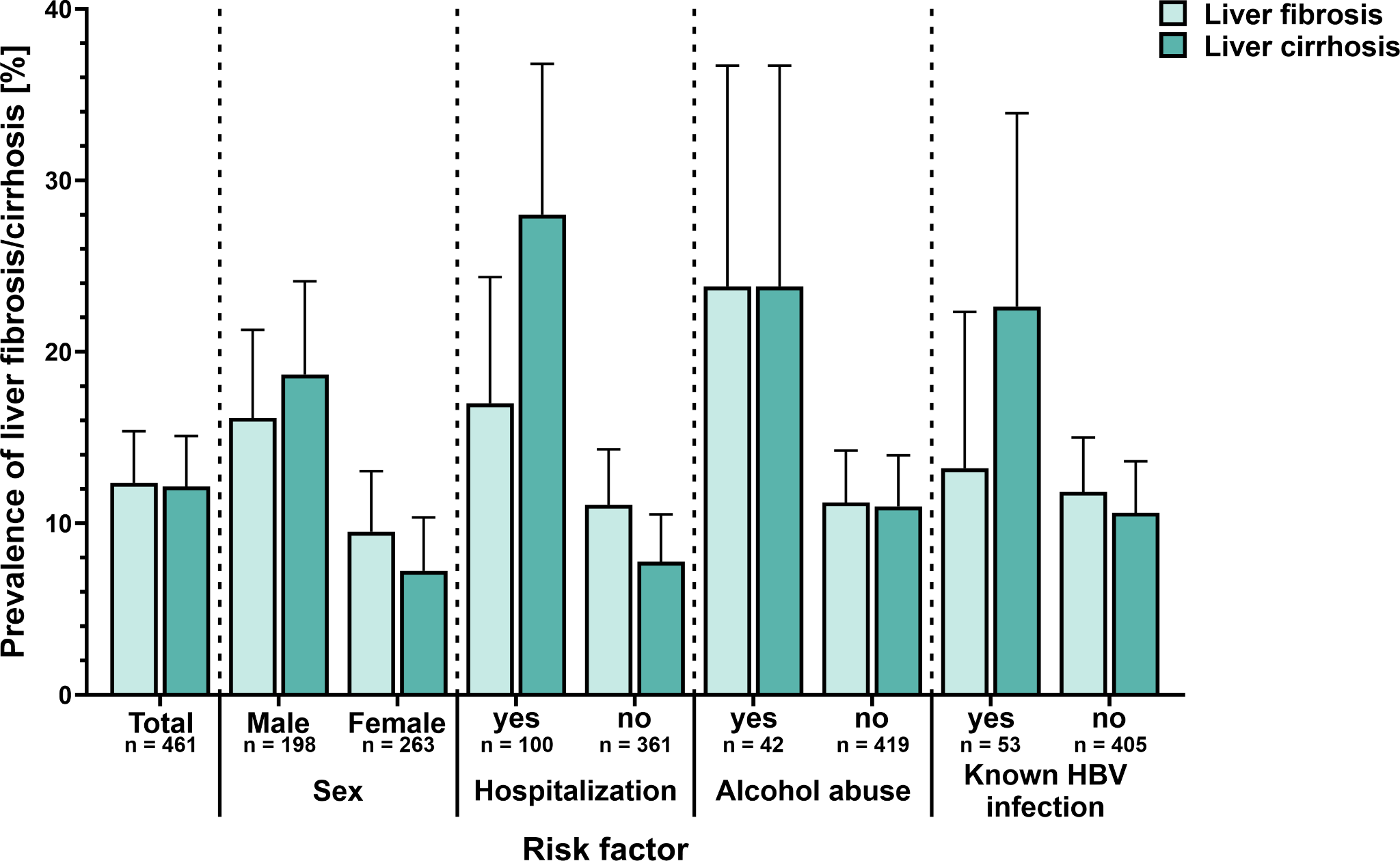


**Figure S1. Prevalence (± 95% CI) of liver fibrosis and liver cirrhosis for selected risk factors.** Risk factors were defined as: known HBV infection (self-reported knowledge about pre-existing HBV infection), alcohol abuse (self-reported current or past alcohol abuse and/or elevated carbohydrate-deficient transferrin (CDT) level).


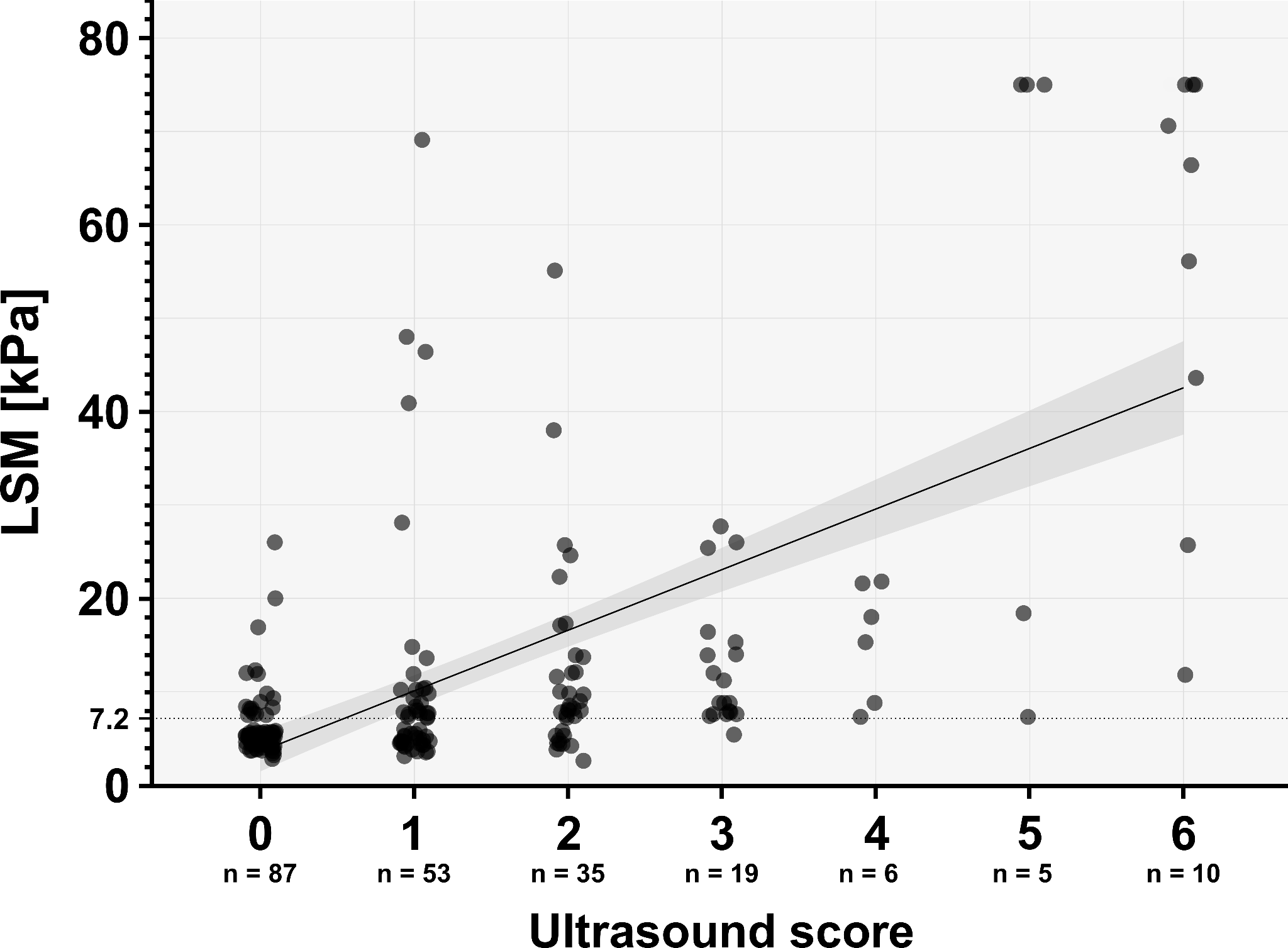


**Figure S2. Correlation of ultrasound score and liver stiffness measurement (LSM) values.** Plot of individual data points as well as a regression line with 95% CI. The cut-off used for LSM values to be considered pathological (≥7.2 kPa) is shown as a dotted line.

**
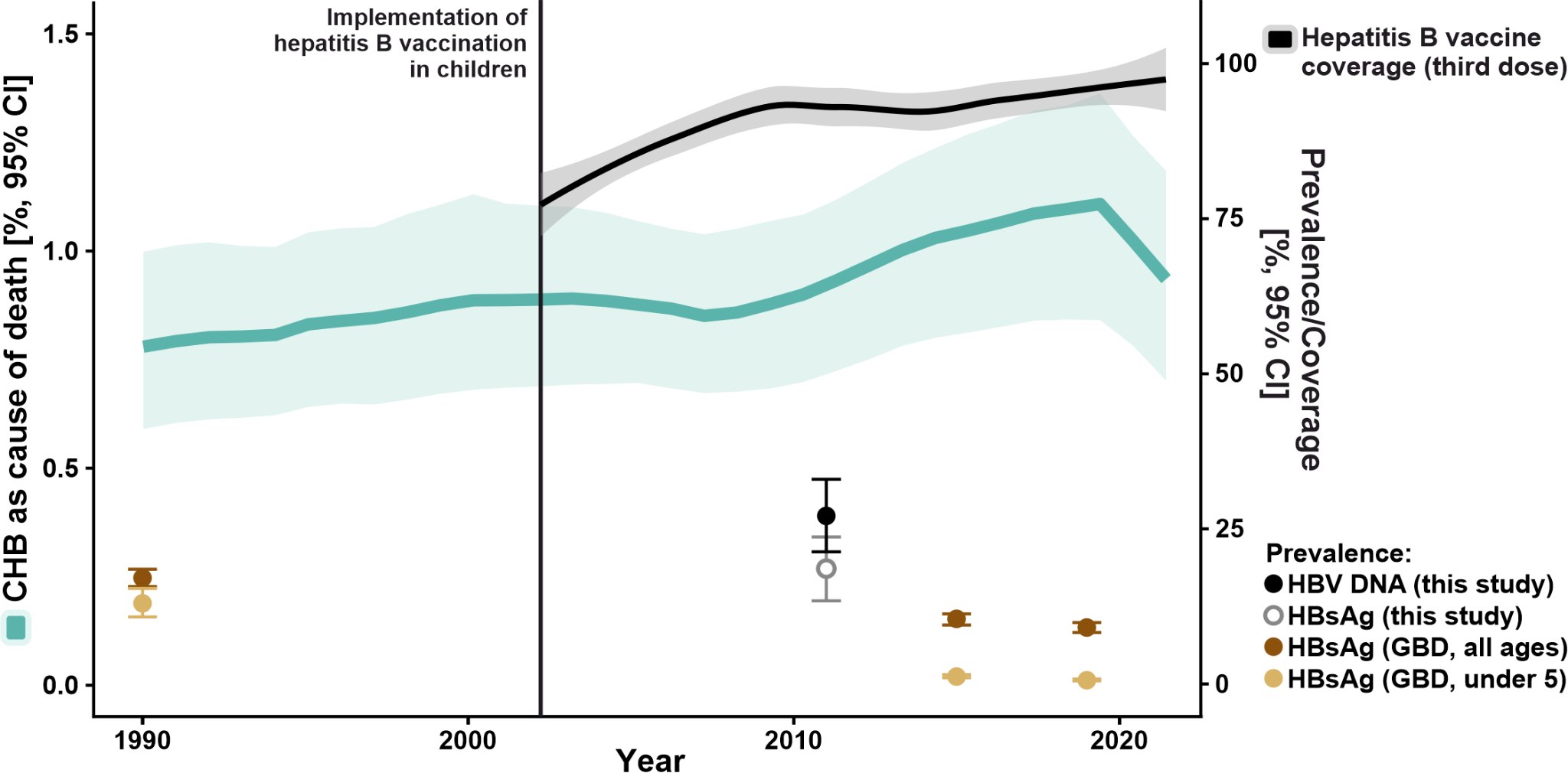
**

**Figure S3. Health statistics on hepatitis B in Ghana, 1990-2021.** Hepatitis B vaccine coverage according to WHO/UNICEF estimates of national immunisation coverage in newborns (WUNIC). HBsAg prevalence of the general population and children under 5 years of age as published by the Global Burden of Disease (GBD) 2019 Hepatitis B Collaborators [1]. Data on chronic hepatitis B (CHB) as cause of death as published by the GBD collaboration [2] (downloaded via [GBD compare](https://vizhub.healthdata.org/gbd-compare/), indicator B.4.1.1 Chronic hepatitis B including cirrhosis).

**SUPPLEMENTARY TABLES**

**Table S1. Primers designed for this study.** Ambiguous nucleotides: Y:T/C, R:G/A, M:A/C, K:G/T, S:G/C, N:A/T/G/C, I:Inosin

| **Primer name** | **Sequence [5’-3’]** | **Polarity** |
| --- | --- | --- |
| HBV-F1398 | CTG GAT TCT GCG CGG GAC GTC CTT | + |
| HBV-R1883 | GGC ACA GCT TGG AGG CTT GAA | - |
| HBV-R1798a | ACC AAT TTA TGC CTG CAG CCT CC | - |
| HBV-R1798b | ATG TAT TTA TGC CTA CAG CCT CC | - |
| HBV-Ghana-S-F | CAG TGG TTC GTC GGG CTT TC | + |
| HBV-R1204 | CAG TGG GGG TTG CGT CAG CAA A | - |
| HBV-Ghana-S-Fnest | CCC CAY TGT CTG GCT TTY AGT | + |
| HBV-F1770 | GTA TTA GGA GGC TGT AGG CAT AAA T | + |
| HBV-R2419 | GCG ACG CGG TGA TTG AGA YCT | - |
| HBV-F1865 | CAA GCC TCC AAG CTG TGC CTT | + |
| HBV-F2814 | GGG TCA CCA TAT TCT TGG GAA CA | + |
| HBV-Ghana-S-R | GTT GGT GAG TGA TTG GAG GTT G | - |
| HBV-Ghana-S-Rnest | CGA ATT TTG GCC AMG ACA CAC | - |
| HBV-Ghana-F-A2 | CAG GCT TTC ACT TTC TCG CCA A | + |
| HBV-Ghana-R-A1 | AGA GGT GAA GCG AAG TGC ACA | - |
| HBV-Ghana-Fnest-A2 | TTT ACC CCG TTG CCC GGC AA | + |
| HBV-Ghana-Rnest-A1 | AAG AGA GGT GCG CCC CGT G | - |
| HBV-Ghana-F-A3 | GGT GTC TTT TGG AGT GTG GAT TC | + |
| HBV-Ghana-R-A4 | ACT GGT GGT CGG GAA AAA ATC C | - |
| HBV-Ghana-Fnest-A3 | CTT AYA GAC CAC CAA ATG CCC CTA | + |
| HBV-Ghana-Rnest-A4 | CAG AGG ATT GGT GGT GGA ATG A | - |
| HDV-F133mod | GAG GAI GAI AAY CCI TGG YTK GG | + |
| HDV-R473 | GAG GAG AAY CCG TGG YTG GGN AA | - |
| HDV-F136 | AGY TGI TTC TTY TTG TTC TCS AG | + |
| HDV-R347 | CCI YTI RGI GGG TTC ACA TCC CCA | - |

**Table S2. Genotypic analysis of hepatitis B virus.** #: numbering according to the ICTV-reference strain of HBV genotype E (GenBank acc. no.: X75657); 1: decreased response to interferon [3], 2: associated with fulminant hepatitis B [4–6], 3: nonsense mutation G1896A (often accompanied by G1899A) leads to loss of HBeAg [7], 4: loss of MHBs, 5: confers limited susceptibility to lamivudine [8], 6: confers full resistance to lamivudine and telbivudine, partial resistance to entecavir [8], 7: loss of conserved N-glycosylation site. BCP: basal core promoter; LSM: liver stiffness measurement; n.a.: not available; OBI: occult hepatitis B infection; RT: reverse transcriptase; WT: wild type; *: stop codon.

| **ID** | **HBV-DNA titer [log_10_ IU/mL]** | **Recovered sequence length [nt]** | **Genome positions covered^#^** | **Genotype** | **HBeAg assay** | **Serotype** | **BCP/preC** | **RT domain resistance** | **S domain escape** | **preS2 domain** | **LSM [kPa]** | **Comments** |
| --- | --- | --- | --- | --- | --- | --- | --- | --- | --- | --- | --- | --- |
| 4 | 2.06 | 464 | 272 - 735 | E | negative | ayw4 | n.a. | none | none | n.a. | 40.9 | OBI |
| 5 | 4.61 | 3,212 | 1 - 3,212 | E | negative | ayw4 | G1757A^1^, A1762T^2^, G1764A^2^, G1896A^3^, G1899A | none | none | M1V^4^ | 5.5 | - |
| 7 | 2.91 | 464 | 272 - 735 | E | negative | ayw4 | n.a. | none | none | n.a. | 4.8 | - |
| 14 | 4.18 | 3,182 | 1- 1,517 / 1,795 - 3,212 | E | negative | ayw4 | G1757A^1^,  A1762T^2^, G1764A^2^, G1896A^3^, G1899A | none | none | M1I^4^, Q13-F22del | 46.4 | - |
| 22 | 1.77 | 400 | 255 - 654 | E | negative | ayw4 | n.a. | none | C69*, G130R, F134L | n.a. | 15.3 | OBI |
| 24 | 9.40 | 464 | 272 - 735 | E | positive | ayw4 | n.a. | none | F134L | n.a. | 70.6 | anti-HCV positive |
| 25 | 7.45 | 514 | 247 - 760 | E | positive | ayw4 | n.a. | none | C69*, S143L, G145R | n.a. | 25.7 | - |
| 29 | 8.82 | 464 | 272 - 735 | E | positive | ayw4 | n.a. | none | none | n.a. | 26.0 | - |
| 30 | 7.67 | 464 | 272 - 735 | E | positive | ayw4 | n.a. | none | none | n.a. | 21.6 | - |
| 32 | 1.48 | 464 | 272 - 735 | E | negative | ayw4 | n.a. | none | none | n.a. | 3.7 | - |
| 33 | 2.1 | 464 | 272 - 735 | E | negative | ayw4 | n.a. | none | none | n.a. | 5.8 | - |
| 37 | 5.23 | 3,212 | 1 -3,212 | E | negative | ayw4 | G1757A^1^, G1899A | none | W201*, L209*^3^ | WT | 10.0 | - |
| 40 | 2.27 | 464 | 272 - 735 | E | negative | ayw4 | n.a. | none | none | n.a. | 9.0 | - |
| 41 | 1.50 | 464 | 272 - 735 | E | negative | ayw4 | n.a. | none | none | n.a. | 8.0 | - |
| 54 | 7.47 | 464 | 272 - 735 | E | positive | ayw4 | n.a. | none | none | n.a. | 69.1 | - |
| 82 | 5.05 | 464 | 272 - 735 | E | positive | ayw4 | n.a. | none | L127P, D144E | n.a. | 6.1 | HIV coinfection |
| 131 | 5.31 | 464 | 272 - 735 | E | positive | ayw4 | n.a. | none | none | n.a. | 16.4 | HIV coinfection |
| 136 | 8.66 | 464 | 272 - 735 | E | negative | ayw4 | n.a. | V173L^5^, L180M^6^ | none | n.a. | 7.2 | HIV coinfection |
| 137 | 8.26 | 464 | 272 - 735 | E | positive | ayw4 | n.a. | V173L^5^, L180M^6^ | none | n.a. | 5.7 | HIV coinfeciton |
| 143 | 4.44 | 3,212 | 1 - 3,212 | E | negative | ayw4 | G1757A^1^, C1766G,  G1896A^3^, G1899A | none | none | WT | 8.2 | HIV coinfection |
| 145 | 8.17 | 3,212 | 1 - 3,212 | E | negative | ayw4 | T1753C, G1757A^1^, G1896A^3^, G1899A | L180M^6^, M204V^6^ | L127P, G145A | WT | 27.7 | HIV coinfection |
| 146 | 7.66 | 3,102 | 1 - 2,170 / 2,280 - 3,212 | E | negative | ayw4 | A1752C, G1757A^1^, G1764A^2^, C1766G, G1896A^3^, G1899A | V173L^5^, L180M^6^, M204V^6^ | none | WT | 13.7 | HIV coinfection |
| 148 | 4.73 | 3,212 | 1 - 3,212 | E | negative | ayw4 | A1752C, G1757A^1^,  G1764A^2^, C1766G, G1896A^3^ | V173L^5^, L180M^6^, M204V^6^ | none | WT | 9.8 | HIV coinfection |
| 184 | 3.65 | 464 | 272 - 735 | E | positive | ayw4 | n.a. | none | none | n.a. | 25.4 | - |
| 189 | 2.98 | 464 | 272 - 735 | E | negative | ayw4 | n.a. | none | none | n.a. | 11.8 | - |
| 212 | 1.55 | 464 | 272 - 735 | E | n.a. | ayw4 | n.a. | L180M^6^ | none | n.a. | 11.2 | - |
| 265 | 2.17 | 464 | 272 - 735 | E | negative | ayw4 | n.a. | none | W182*W | n.a. | 75.0 | - |
| 269 | 3.31 | 3,052 | 1- 1,795 / 1,841 - 2,013 / 2,044 - 2,144 / 2,229 - 3,212 | E | negative | ayw4 | T1754G, A1755T, A1762T^2^, G1763C,  G1764A^2^, C1766T,  T1767A, T1768C | none | M1I, W36*, W182*W | M1I^4^ | 8.4 | OBI |
| 277 | 4.07 | 2,915 | 1- 1,518 / 1,796 - 3,212 | E | negative | ayw4 | G1896A^3^, G1899A | none | none | R16-F22del | 66.4 | - |
| 368 | 7.14 | 464 | 272 - 735 | E | positive | ayw4 | n.a. | none | G145A | n.a. | 75.0 | - |
| 395 | 7.75 | 3,209 | 1- 52 / 56 - 3,212 | E | negative | ayw4 | A1762T^2^, G1764A^2^, G1899A | none | M133I, F134L | M1V^4^, T6K^7^, F22del | 56.1 | HIV coinfection |
| 435 | 1.95 | 464 | 272 - 735 | E | negative | ayw4 | n.a. | none | none | n.a. | 4.4 | OBI |
| 446 | 4.31 | 2,901 | 1 - 1,794 / 1,897 - 2,038 / 2,248 - 3,212 | E | negative | ayw4 | T1754A, A1755G,  G1756T, G1757T,  T1758A, T1759A,  A1760T, A1761C, G1763T, G1764T,  T1765A | none | none | T6K^7^ | 75.0 | HIV and HDV coinfection |
| 470 | 3.59 | 2,936 | 1 - 1517 / 1,795 - 3,212 | E | negative | ayw4 | G1899A | none | P120T, T126I, L127P, A128V, P142L, D144A, G145A | WT | 8.9 | OBI |
| 504 | 2.29 | 464 | 272 - 735 | E | negative | ayr | n.a. | none | P120A, M133I, F134S, K160R | n.a. | 4.3 | OBI |
| 518 | 7.48 | 464 | 272 - 735 | A1 | positive | ayw1 | n.a. | none | L127P | n.a. | 75.0 | anti-HCV positive |

**Supplementary references**

[1] GBD-2019-Hepatitis-B-Collaborators, Sheena BS, Hiebert L, et al. Global, regional, and national burden of hepatitis B, 1990–2019: a systematic analysis for the Global Burden of Disease Study 2019. Lancet Gastroenterol Hepatol 2022;7:796–829. <https://doi.org/10.1016/s2468-1253(22)00124-8>.

[2] GBD-2021-Causes-of-Death-Collaborators, Naghavi M, Ong KL, et al. Global burden of 288 causes of death and life expectancy decomposition in 204 countries and territories and 811 subnational locations, 1990–2021: a systematic analysis for the Global Burden of Disease Study 2021. Lancet 2024;403:2100–32. <https://doi.org/10.1016/s0140-6736(24)00367-2>.

[3] Erhardt A, Reineke U, Blondin D, et al. Mutations of the core promoter and response to interferon treatment in chronic replicative hepatitis B. Hepatology 2000;31:716–25. <https://doi.org/10.1002/hep.510310323>.

[4] Hasegawa K, Huang J, Rogers SA, et al. Enhanced replication of a hepatitis B virus mutant associated with an epidemic of fulminant hepatitis. J Virol 1994;68:1651–9. <https://doi.org/10.1128/jvi.68.3.1651-1659.1994>.

[5] Sato S, Suzuki K, Akahane Y, et al. Hepatitis B Virus Strains with Mutations in the Core Promoter in Patients with Fulminant Hepatitis. Ann Intern Med 1995;122:241. <https://doi.org/10.7326/0003-4819-122-4-199502150-00001>.

[6] Friedt M, Gerner P, Lausch E, et al. Mutations in the basic core promotor and the precore region of hepatitis b virus and their selection in children with fulminant and chronic hepatitis B. Hepatology 1999;29:1252–8. <https://doi.org/10.1002/hep.510290418>.

[7] Kumar R. Review on hepatitis B virus precore/core promoter mutations and their correlation with genotypes and liver disease severity. World J Hepatol 2022;14:708–18. <https://doi.org/10.4254/wjh.v14.i4.708>.
